# A comprehensive dataset of surface deformation satellite maps of the valley of Toluca, Mexico

**DOI:** 10.1016/j.dib.2023.109733

**Published:** 2023-10-28

**Authors:** Iván Francisco-Valencia, Roberto Alejo-Eluterio, Javier Salas-Garcia, Rosa María Valdovinos-Rosas, Everardo Granda-Gutiérrez

**Affiliations:** a Instituto Tecnológico de Toluca, Av. Tecnológico S/N. Colonia Agrícola Bellavista C.P. 52149, Metepec, Estado de México, Mexico; b Facultad de Ingeniería, Universidad Autónoma del Estado de México, Cerro de Coatepec S/N Ciudad Universitaria C.P. 50100. Toluca, Estado de México, Mexico; c Centro Universitario UAEM Atlacomulco, Universidad Autónoma del Estado de México, Autopista Toluca-Atlacomulco km 60, C.P. 50400, Atlacomulco, Estado de México, Mexico

**Keywords:** Land monitoring, Interferogram, DInSAR, Sentinel 1, Coherence interferogram

## Abstract

This work presents a substantial compilation of ground deformation maps portraying the dynamic evolution of the Valley of Toluca (VT) in Mexico. The dataset comprises a repository of 1121 BEAM-DIMAP formated maps obtained by the Differential Interferometric Synthetic Aperture Radar (DInSAR) technique. Leveraging satellite image pairs from the Sentinel 1-A and Sentinel 1-B satellites, the dataset spans intervals of 1, 3, 6, and 12 months between each image acquisition and covers a panoramic timeframe from October 2014 to December 2022. This compilation provides an in-depth chronicle of the VT's ground transformations over a span of eight years that could be of interest to various disciplines. To enhance the dataset's robustness, a supplementary comma-separated values (CSV) dataset includes the coherence statistics from the satellite image pairs, substantiating the precision and dependability of the ground deformation maps presented herein.

Specifications Table

**Table:** 

Subject	Earth-Surface Processes.
Specific subject area	Environmental Geology.
Data format	Raw, Analyzed.
Type of data	Table, Image, Figure
Data collection	The deformation maps were generated from 240 satellite images from the Sentinel 1-A and Sentinel 1-B missions. These images span the period from October 2014 to December 2022 and have the following characteristics: • Product Type: Level-1 Single Look Complex (SLC) • Acquisition Mode: Interferometric Wide (IW), with a swath width of 250 km at a spatial resolution of 5 m by 20 m. • Orbit: Descending. • Polarization: Single VV and Dual VV+VH. The images were sourced from the Alaska Satellite Facility Data Search Vertex [1] and processed using the Sentinel Application Platform (SNAP) software.
Data source location	The data correspond to remote sensing images of the Valley of Toluca (VT) in Mexico (GPS coordinates: 19° 03’ to 19°35’ north latitude and 99°19’ to 99°54’ west longitude).
Data accessibility	Repository name: Zenodo Data identification number: 10.5281/zenodo.8311666 Direct URL to data: https://doi.org/10.5281/zenodo.8311666

## 1 Value of the Data

• The DInSAR technique used for deformation mapping is computationally intensive, and this dataset offers ready-made deformation maps, which are invaluable for researchers, especially those in soil studies using Machine Learning or Big Data techniques. The dataset spans eight years of ground monitoring, making it beneficial for studying long-term phenomena like subsidence, landslides, or volcanic activity. Moreover, the maps are presented in BEAM-DIMAP format, ensuring reusability in SNAP software and other platforms.
• This dataset is valuable for researchers in hydrogeology, remote sensing, artificial intelligence, and related fields, as well as governmental institutions and organizations involved in risk management and territorial planning.
• The extensive temporal coverage and detailed information in this dataset provide a foundation for future research, enabling temporal comparisons and in-depth analyses.
• Accompanied by a CSV file, the dataset includes statistical data for each map, enhancing the visual information and facilitating various statistical analyses.
• Covering the Valley of Toluca, this dataset offers a comprehensive view of regional deformation over time, crucial for localized studies and interventions.
• Offering deformation maps from the Sentinel 1-A and Sentinel 1-B missions, this dataset significantly contributes to the remote sensing community, providing a reliable source of information for various applications.

## 2 Data Description

A total of 1121 satellite maps are presented in BEAM-DIMAP file format (compressed in zip format). Each one comprises a single band containing terrain deformation information. These maps can be utilized within the SNAP software (the same platform through which they were processed) or imported into a usual geographic information software (GIS), such as the proprietary ArcGIS or the Open Source QGIS (licensed under the GNU General Public License).

Each BEAM-DIMAP file adheres to the following nomenclature:fmaster_fslave_Stack_Ifg_Deb_DInSAR_ML_Flt_Sub_Unw_Disp_TC.dim

where:

fmaster: Represents the acquisition date of the Master image.

fslave: Corresponds to the acquisition date of the Slave image.

The remaining components of the filename correspond to the sequence of steps undertaken in the DInSAR technique for obtaining the deformation map. Please refer to the “**Production of deformation maps based on remote sensing images**” section for a detailed breakdown of these steps.

**Fig. 1:**
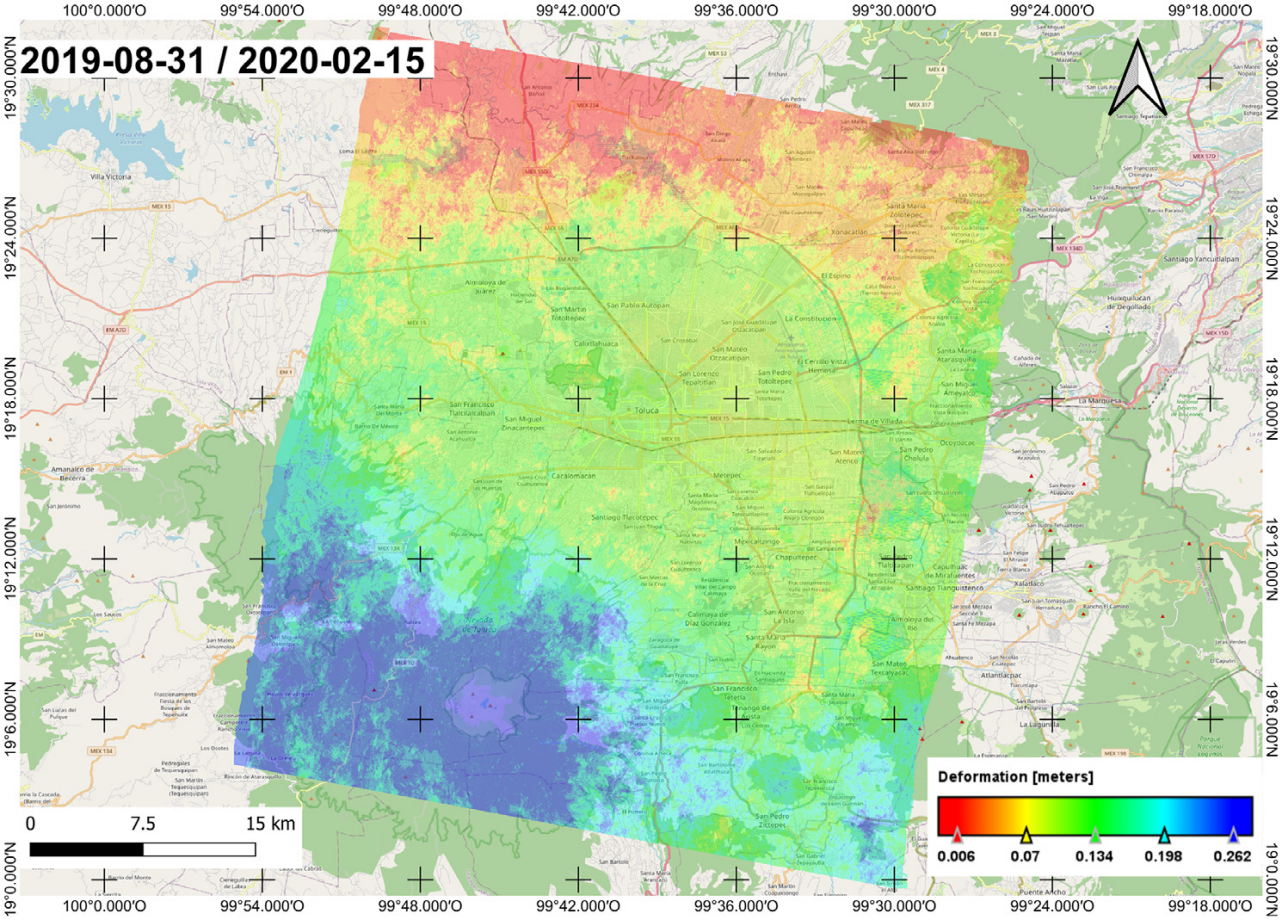
Deformation map of the VT for August 31st, 2019, to February 15th, 2020, imported and visualized in QGIS.

As a result of the process, terrain deformation maps for the VT were derived. For example, Fig. 1 illustrates the deformation map from August 31st, 2019, to February 15th, 2020, imported and processed within the QGIS software to get a visual representation. The complete dataset of 1121 processed deformation maps and their associated CSV file can be retrieved from [2].

Furthermore, a CSV file is provided (stats.csv), which display statistical data for each map. The columns within the table represent the following information:

• Master Image Acquisition Date
• Slave Image Acquisition Date
• Average Deformation in Meters (disp_avg)
• Maximum Deformation in Meters (disp_max)
• Minimum Deformation in Meters (disp_min)
• Median Deformation in Meters (disp_med)
• Average Coherence between Master and Slave Images (disp_avg)
• Maximum Coherence between Master and Slave Images (disp_max)
• Minimum Coherence between Master and Slave Images (disp_min)
• Median Coherence between Master and Slave Images (disp_med)

## 3 Experimental Design, Materials and Methods

### 3.1 Area of interest

**Fig. 2:**
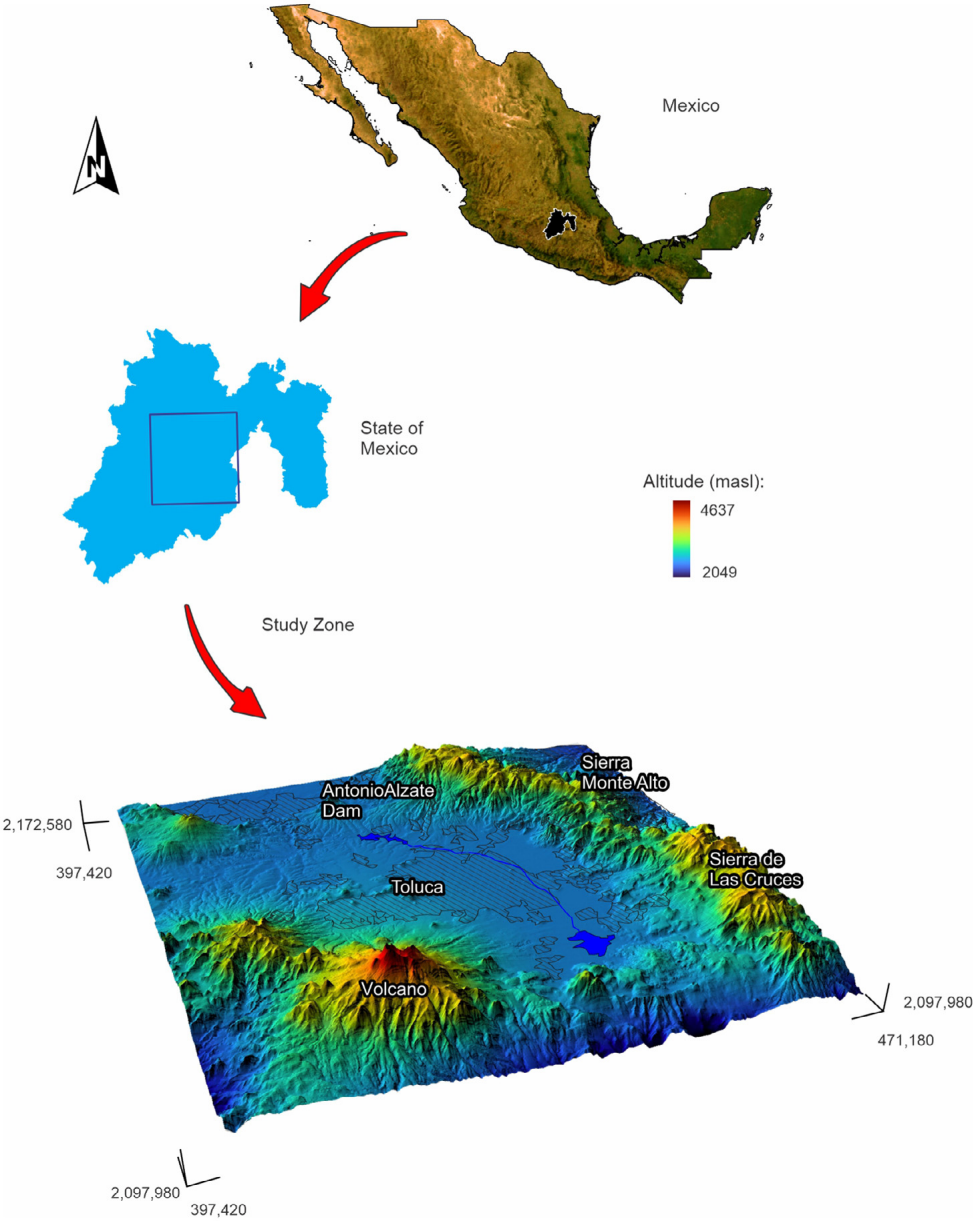
Location of the Valley of Toluca, Mexico.

According to [3], the VT is situated within the coordinates of 19°03’ to 19°35’ north latitude and 99°19’ to 99°54’ west longitude. This geographic expanse is located inside the State of Mexico, positioned at the central portion of both Mexico and the Trans-Mexican Volcanic Belt. Physically bounded by three mountain ranges: Monte Alto, de las Cruces, and Tenango (see Fig. 2), the VT region is in the heart of this geological context.

The maximum altitude point of the VT is the "Nevado de Toluca or Xinantécatl" volcano, soaring to an impressive 4340 m above sea level. In contrast, the rest of the valley boasts an average elevation of 2640 m above sea level [4]. The climate enveloping the VT assumes two distinct guises: a temperate subhumid variant and a semi-cold subhumid counterpart. Within this climatic dichotomy, the annual mean temperature oscillates between −3 °C and 14 °C, while the yearly precipitation levels are between 800 and 1200 mm [4]. Regarding the soil composition, the terrain predominantly presents a mixture of soil types, prominently including Andosols, reflective of volcanic zones, along with Feozems, Vertisols, Luvisols, and Cambisols [4].

One of the most populated areas in Mexico, reaching 2157,640 inhabitants by 2020 [5], is located in the VT which is constituted by the Toluca Valley Metropolitan Area, that consider the municipalities of Almoloya de Juárez, Calimaya, Chapultepec, Lerma, Metepec, Mexicaltzingo, Ocoyoacac, Otzolotepec, Rayón, San Antonio la Isla, San Mateo Atenco, Toluca, Xonacatlán and Zinacantepec [3].

In the VT region, economic activities are categorized as follows [4]:

• **Primary Sector:** Seasonal agriculture and extensive livestock farming.
• **Secondary Sector:** Metallurgical, textile, chemical, petrochemical, glass and derivatives, rubber and plastics industries, electricity generation, mining industry, and construction industry.
• **Tertiary Sector:** Food trade, waste management, and hotel administration.

Regarding soil-related phenomena, the VT presents various types of risks, primarily [6]:

• **Volcanic Hazards:** Due to its proximity to the Nevado de Toluca volcano.
• **Seismic Activity:** Due to its proximity to the states of Michoacán, Oaxaca, and Guerrero, which experience the highest frequency of earthquakes in the country.
• **Soil Fractures and Subsidence:** In the municipality of Toluca, over thirty fracture points have been identified [7,8].
• **Landslides:** Risks of slope instability and landslides.
• **Erosion:** Vulnerabilities attributed to soil erosion due to excessive deforestation and changes in soil use.

### 3.2 Production of deformation maps based on remote sensing images

The deformation maps were generated using the DInSAR technique, which is based on analyzing the phase difference between two radar images to determine millimetric-level changes in the land's surface. To accomplish the dataset, 240 radar images were obtained from the Sentinel 1-A and Sentinel 1-B missions in an 8-year monitoring period of the VT, from October 8th, 2014, to December 23rd, 2022. The IW swath mode is the main acquisition mode over land in the Sentinel 1 missions, which uses three sub-swaths. In this work, only radar images where the VT falls within a single sub-swath were selected.

Using the 240 radar images, pairs of images were generated following the following criteria:

• All images from a given month ‘x’ were paired with images from the subsequent month ‘x+1′.
• All images from a given month ‘x’ were paired with images from ‘x+3’ months.
• All images from a given month ‘x’ were paired with images from ‘x+6’ months.
• All images from a given month ‘x’ were paired with images from ‘x+12’ months.

**Fig. 3:**
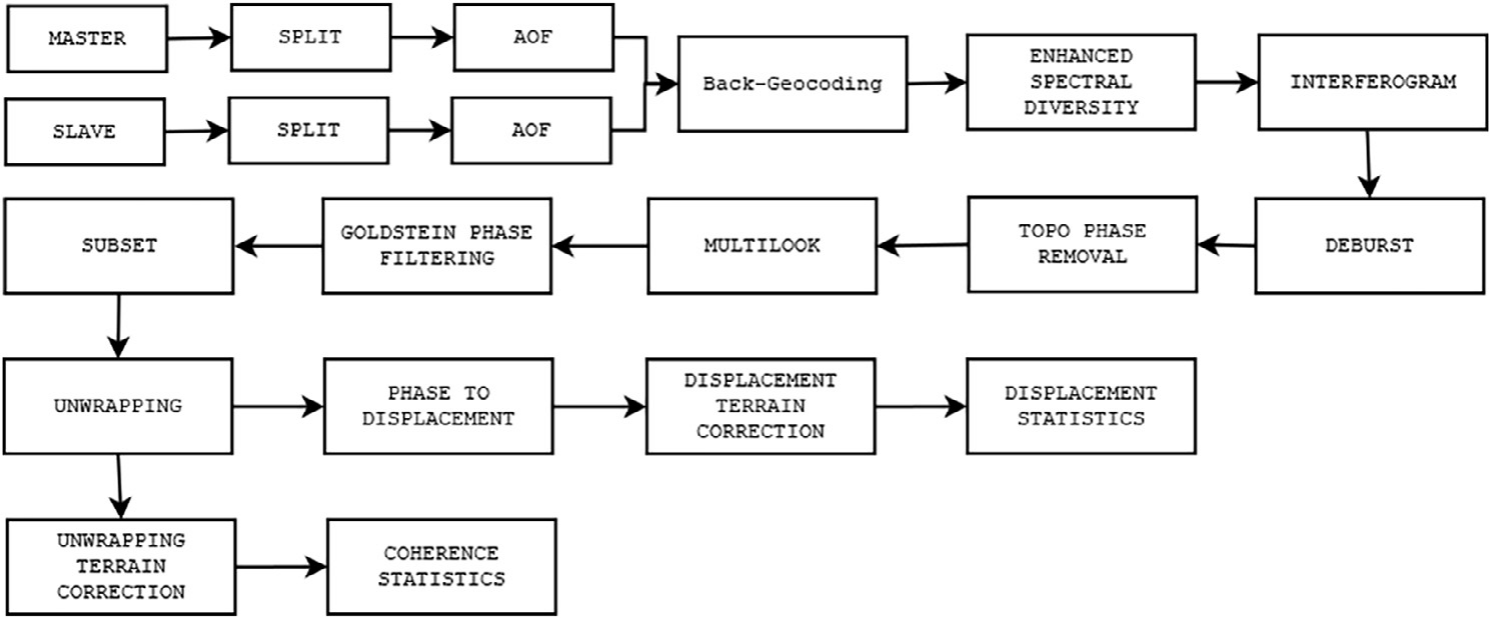
Steps of the DInSAR technique used for the generation of deformation maps.

From that, a total of 2169 pairs of images were obtained, which were subsequently processed using the DInSAR technique. The steps of this technique are illustrated in Fig. 3 and explained as follows:

• **Master:** Refers to the reference image.
• **Slave:** The image acquired by the Sentinel satellite at a later date than the Master.
• **Split:** This step involves selecting the sub-swath and bursts that encompass the VT.
• **Apply Orbit File (AOF):** The original radar images from the Master and Slave did not contain information about the satellite's position when they were acquired by the Sentinel 1-A and Sentinel 1-B missions [9]. This information is crucial for the subsequent steps. Consequently, positional information is retrieved and associated with the Master and Slave images in this step.
• **Back-Geocoding:** In this step, the Master and Slave images are co-registered to ensure that both are geographically aligned. The Shuttle Radar Topography Mission (SRTM) digital elevation model was utilized for this purpose [9].
• **Enhanced Spectral Diversity:** This step is applied to enhance the quality of co-registration achieved in the previous step.
• **Interferogram:** The interferogram represents the phase variation between the two images and is related to differences in object distances [9]. The interferogram is obtained by cross-multiplying the Master image with the conjugate of the Slave image [9]. Additionally, the Coherence band is calculated, which serve as a quality indicator. It measures how similar are the pixels between the Slave and Master images on a scale of 0 to 1. A value close to 0 indicates low correlation and high noise presence, while a value near 1 indicates high correlation and low noise presence [9,10].
• **Deburst:** Removes the seamlines between the bursts of Master and Slave images.
• **Topo Phase Removal:** In this step, the topographic phase and elements not contributing to obtaining terrain deformation are eliminated [9]. Like the Back-Geocoding stage, the SRTM elevation model is used in this step also.
• **Multilook:** This step is applied to reduce noise in the interferogram from the previous step. It involves obtaining the pixel mean in each interferogram direction [9].
• **Goldstein Phase Filtering:** The Goldstein Phase Filtering technique is applied to reduce noise and enhance precision [9].
• **Subset:** In this step, the area of interest in the interferogram is narrowed down to only the VT, aiming to reduce processing time for the subsequent steps.
• **Unwrapping:** In this step, the interferometric phase, which is in a 2π scale, is unwrapped to relate it to topographic height [9,10]. Unlike the previous steps, this process uses the SNAPHU software version 1.4.2 for Windows.
• **Phase to Displacement:** Once the interferometric phase is unwrapped, it is converted into heights in meters, where positive values indicate elevation and negative values indicate subsidence [9].
• **Unwrapping/Displacement Terrain Correction:** In this step, the SRTM digital elevation model is again employed to correct the result from the previous step geometrically. The final deformation map is obtained in the case of the Displacement Terrain Correction step.
• **Coherence/Displacement Statistics:** In this step, coherence/deformation statistics are computed, including maximum, minimum, average, and mean values.

When the processing step is accomplished, the BEAM-DIMAP format images can be visualized and further processed, if needed, using geographic information software. Additionally, the CSV data files can be employed to enhance visual information and facilitate various statistical analyses concerning the VT area.

## 4 Limitations

Due to the high quantity of space required for store the 2169 deformation maps of the Valley of Toluca, the following considerations were made:

• The area of interest has been constrained between coordinates 19°00’ and 19°30’ north latitude and 99°24’ and 99°54’ west longitude, encompassing most of the region.
• Only 1121 deformation maps, whose average coherence is greater than 0.299, are available in the repository.
• The ‘stats.csv’ file has not been modified and still contains the statistics of 2169 maps.

However, the complete 2169 maps that are outcome of the Goldstein Phase Filtering step, which covers the entire Valley, is available upon request via the corresponding author's email.

## Ethics Statement

The authors have read and followed the ethical requirements for publication in Data in Brief and confirm that the present work does not involve human subjects, animal experiments, or any data collected from social media platforms.

## CRediT authorship contribution statement

**Iván Francisco-Valencia:** Conceptualization, Investigation, Software, Writing – original draft. **Roberto Alejo-Eluterio:** Conceptualization, Methodology, Writing – review & editing, Supervision. **Javier Salas-Garcia:** Methodology, Writing – review & editing. **Rosa María Valdovinos-Rosas:** Methodology, Writing – review & editing. **Everardo Granda-Gutiérrez:** Writing – review & editing.

## Data Availability

A Comprehensive Dataset of Surface Deformation Satellite Maps of The Valley of Toluca, Mexico (Original data) (Zenodo)
